# Characterization and functional analysis of *Toxoplasma* Golgi-associated proteins identified by proximity labeling

**DOI:** 10.1128/mbio.02380-24

**Published:** 2024-09-30

**Authors:** Rebecca R. Pasquarelli, Justin J. Quan, Emily S. Cheng, Vivian Yang, Timmie A. Britton, Jihui Sha, James A. Wohlschlegel, Peter J. Bradley

**Affiliations:** 1 Molecular Biology Institute, University of California, Los Angeles, California, USA; 2 Department of Microbiology, Immunology, and Molecular Genetics, University of California, Los Angeles, California, USA; 3 Department of Biological Chemistry and Institute of Genomics and Proteomics, University of California, Los Angeles, California, USA; Albert Einstein College of Medicine, Bronx, New York, USA

**Keywords:** *Toxoplasma gondii*, vesicular trafficking, apicomplexan parasites, protein trafficking, Golgi apparatus, ER exit sites, secretory pathway

## Abstract

*Toxoplasma gondii* possesses a highly polarized secretory pathway that contains both broadly conserved eukaryotic organelles and unique apicomplexan organelles, which play essential roles in the parasite’s lytic cycle. As in other eukaryotes, the *T. gondii* Golgi apparatus sorts and modifies proteins prior to their distribution to downstream organelles. Many of the typical trafficking factors found involved in these processes are missing from apicomplexan genomes, suggesting that these parasites have evolved unique proteins to fill these roles. Here, we identify a Golgi-localizing protein (ULP1), which is structurally similar to the eukaryotic trafficking factor p115/Uso1. We demonstrate that depletion of ULP1 leads to a dramatic reduction in parasite fitness that is the result of defects in microneme secretion, invasion, replication, and egress. Using ULP1 as bait for TurboID proximity labeling and immunoprecipitation, we identify 11 more Golgi-associated proteins and demonstrate that ULP1 interacts with the *T. gondii*-conserved oligomeric Golgi (COG) complex. These proteins include both conserved trafficking factors and parasite-specific proteins. Using a conditional knockdown approach, we assess the effect of each of these 11 proteins on parasite fitness. Together, this work reveals a diverse set of *T. gondii* Golgi-associated proteins that play distinct roles in the secretory pathway. As several of these proteins are absent outside of the Apicomplexa, they represent potential targets for the development of novel therapeutics against these parasites.

**IMPORTANCE:**

Apicomplexan parasites such as *Toxoplasma gondii* infect a large percentage of the world’s population and cause substantial human disease. These widespread pathogens use specialized secretory organelles to infect their host cells, modulate host cell functions, and cause disease. While the functions of the secretory organelles are now better understood, the Golgi apparatus of the parasite remains largely unexplored, particularly regarding parasite-specific innovations that may help direct traffic intracellularly. In this work, we characterize ULP1, a protein that is unique to parasites but shares structural similarity to the eukaryotic trafficking factor p115/Uso1. We show that ULP1 plays an important role in parasite fitness and demonstrate that it interacts with the conserved oligomeric Golgi (COG) complex. We then use ULP1 proximity labeling to identify 11 additional Golgi-associated proteins, which we functionally analyze via conditional knockdown. This work expands our knowledge of the *Toxoplasma* Golgi apparatus and identifies potential targets for therapeutic intervention.

## INTRODUCTION

*Toxoplasma gondii* is an obligate intracellular parasite in the phylum Apicomplexa. This phylum includes parasites of both human and veterinary medical importance including *Plasmodium* spp. (malaria), *Cryptosporidium* spp. (diarrheal disease), *Eimeria* spp. (chicken coccidiosis), and *Neospora caninum* (neosporosis) (1–5). Approximately one-third of the global human population is chronically infected with *T. gondii* (6). While infection is usually asymptomatic in healthy individuals, the parasite can cause severe or fatal disease in immunocompromised people and congenitally infected neonates (7–9). While treatments exist that can suppress the acute infection, they are unable to clear the parasite completely resulting in lifelong chronic infection (8). A deeper understanding of apicomplexan biology is needed to inform the discovery of novel parasite-specific therapeutics.

Apicomplexan parasites possess a unique and highly polarized secretory pathway, which plays an essential role in their life cycles (10, 11). The endomembrane system of these parasites includes the endoplasmic reticulum (ER) and a Golgi apparatus, which is composed of a single stack of three to five cisternae, as well as several apicomplexan-specific organelles including the rhoptries, micronemes, dense granules, and inner membrane complex (IMC) (12, 13). During parasite replication, these organelles are either recycled from the maternal parasite or are formed *de novo* from vesicles, which bud from the Golgi and are trafficked through the secretory pathway to their destinations (14, 15). The secretory pathway also delivers cargo to the plant-like vacuole (PLVAC), endosome-like compartment (ELC), and a non-photosynthetic plastid called the apicoplast (16–19). As each of these organelles is essential, correct protein sorting is important for the survival of the parasite.

Recent studies have revealed a surprising intersection between *T. gondii*’s endocytic and exocytic pathways. Several Rabs, which are components of the endosomal system in most eukaryotes, such as Rab5a and Rab7, have instead been repurposed as secretory trafficking proteins that reside in the ELC. The PLVAC, a hydrolytic compartment analogous to the lysosome, has been implicated in multiple roles such as autophagy, degradation of host-derived proteins, and proteolytic processing of proteins that are trafficked to the micronemes and rhoptries (17, 20, 21). Disruption of proteins that localize to the PLVAC and ELC or proteins that are typically involved in endosomal trafficking has been shown to cause defects in the biogenesis of rhoptries and micronemes (11, 17, 22–24). Furthermore, endolysosomal proteases have been shown to proteolytically process immature ROP and MIC proteins (17, 20, 21). Together, this evidence supports the convergence of endocytic and exocytic pathways in *T. gondii*.

Several key trafficking factors that play critical roles in protein trafficking in most eukaryotes are missing in apicomplexan genomes. Specifically, *T. gondii* and other apicomplexans lack most components of the Endosomal Sorting Complexes Required for Transport (ESCRT) and the Golgi-localized Gamma adaptin ear-containing ARF-binding (GGA) proteins (11, 25, 26). Another family of proteins, which was previously thought to be reduced in *T. gondii*, is the conserved oligomeric Golgi (COG) complex proteins (25). The COG complex is an eight-subunit Golgi tethering complex, which is critical for recycling of vesicles containing Golgi-resident enzymes, such as glycosyltransferases, from the endosomes back to the Golgi (27, 28). Finally, the eukaryotic trafficking factor p115/Uso1 appears to be missing from the genomes of apicomplexan parasites, despite the fact that it plays essential roles in anterograde transport, COPI vesicle tethering, and SNARE complex assembly in other systems (29–34). This limited repertoire of classical protein trafficking machinery suggests that apicomplexans may have evolved their own specialized proteins and pathways to facilitate these functions.

Here, we report the identification of a parasite-specific Golgi protein, ULP1, which contains structural features at its N-terminus that are similar to the eukaryotic tethering factor p115/Uso1. We show that loss of ULP1 results in defects in microneme secretion, invasion, replication, and egress. We then use TurboID proximity labeling and immunoprecipitation to show that ULP1 interacts with the *T. gondii* conserved oligomeric Golgi (COG) complex and identify 11 previously uncharacterized Golgi-associated proteins. Finally, we analyze the orthology of each protein and use a conditional knockdown approach to assess their impact on parasite fitness, revealing several essential proteins and several more parasite-specific proteins.

## RESULTS

### TGGT1_289120 is a parasite-specific Golgi protein that is important for parasite fitness

**Fig 1:**
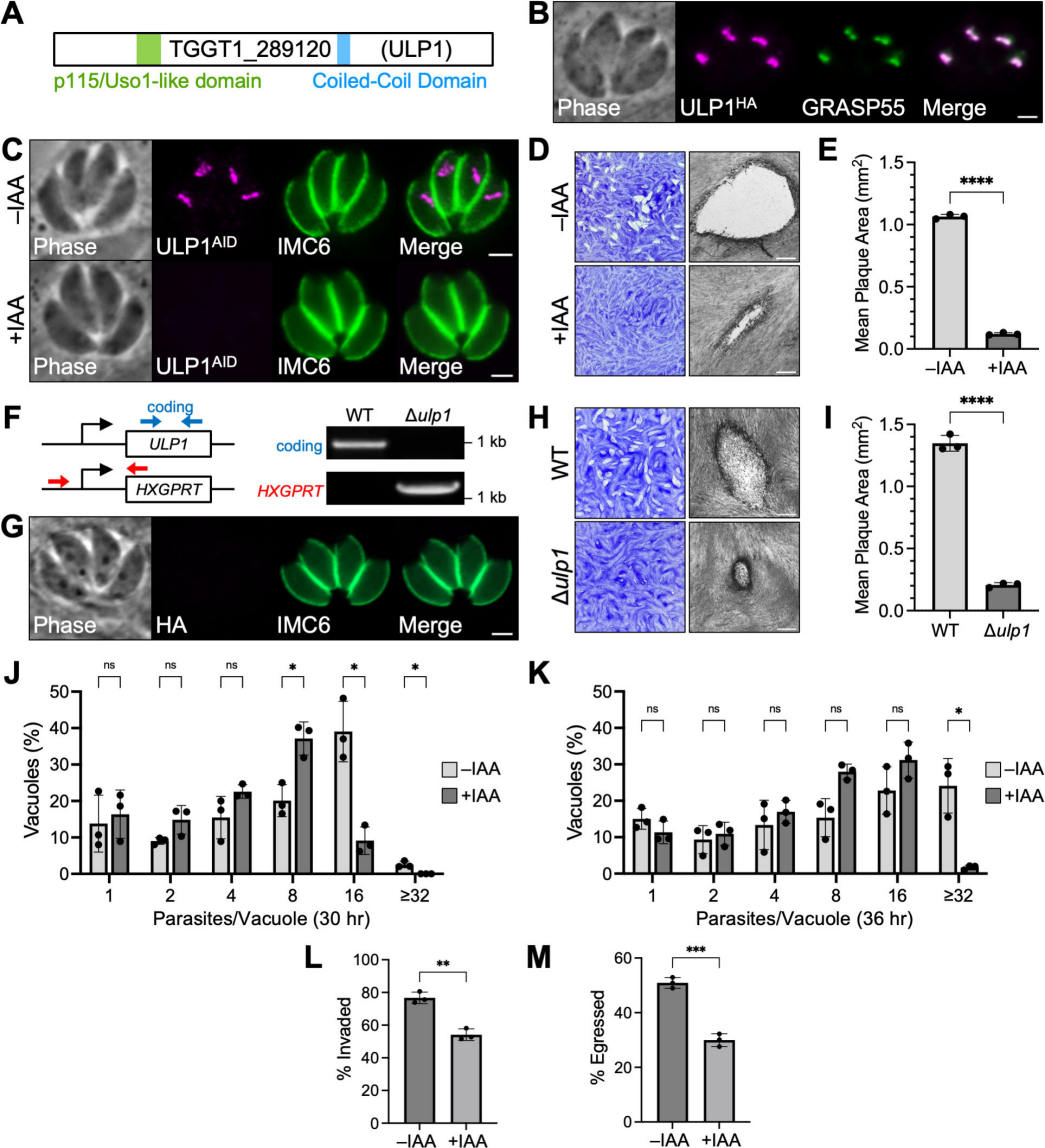
ULP1 is a Golgi-localizing protein that is important for parasite fitness. (**A**) Gene model of TGGT1_289120 (ULP1) showing its p115/Uso1-like domain and predicted CC domain. (**B**) Immunofluorescence assay (IFA) showing that ULP1 colocalizes with the Golgi apparatus marker GRASP55-YFP. Magenta, anti-HA detecting ULP1^3xHA^; green, GRASP55-YFP. (**C**) IFA showing that ULP1^AID^ localizes normally and is depleted after 24 hours of indoleacetic acid (IAA) treatment. Magenta, anti-HA detecting ULP1^AID^; green, anti-IMC6. (**D**) Plaque assay for ULP1^AID^ parasites ±IAA shows that depletion of ULP1 results in a severe reduction in overall lytic ability. (**E**) Quantification of plaque size for plaque assays shown in panel D. Signiﬁcance was determined using a two-tailed t test (****P < 0.0001). (**F**) PCR verification for genomic DNA of wild-type (WT) and Δ*ulp1* parasites. Diagram indicates the binding location of primers used to amplify the ULP1 coding sequencing (blue arrows) and the site of recombination for the knockout (red arrows). (**G**) IFA of Δ*ulp1* parasites confirms loss of ULP1^3xHA^ signal. Magenta, anti-HA; green, anti-IMC6. Scale bars for IFAs = 2 µm. (**H**) Plaque assays of WT and Δ*ulp1* parasites. Scale bars for plaque assays = 0.5 mm. (**I**) Quantification of plaque size for plaque assays shown in panel H. Statistical significance was determined using a two-tailed t test (****P < 0.0001). (**J**) Quantification of the number of parasites per vacuole for ULP1^AID^ parasites treated with IAA or vehicle control after 30 hours of growth. Significance was determined using multiple two-tailed t tests (*P < 0.05; ns = not significant). (**K**) Quantification of the number of parasites per vacuole for ULP1^AID^ parasites treated with IAA or vehicle control after 36 hours of growth. Significance was determined using multiple two-tailed t tests (*P < 0.05; ns = not significant). (**L**) Quantification of invasion for ULP1^AID^ parasites treated with IAA or vehicle control. Significance was determined using a two-tailed t test (**P < 0.01). (**M**) Quantification of egress induced by calcium ionophore for ULP1^AID^ parasites treated with IAA or vehicle control. Significance was determined using a two-tailed t test (***P < 0.001).

We identified a 290-kDa hypothetical protein with the gene ID TGGT1_289120 during our previous proximity labeling experiments using IMC proteins as bait (35). BLASTp analysis revealed that TGGT1_289120 has orthologs in *Hammondia*, *Neospora*, *Besnoitia*, *Cystoisospora*, and *Sarcocystis* but appears to be absent from other apicomplexan parasites and higher eukaryotes (36). Secondary structure analysis using Phyre2 revealed that residues 495–647 of TGGT1_289120 contain structural homology to the globular head domain of p115/Uso1, a membrane-tethering protein conserved in eukaryotes (37, 38). InterPro also identified an armadillo (ARM)-like domain within this region, and DeepCoil2 predicted a coiled-coil (CC) domain within residues 1736–1812 (Fig. 1A) (39, 40). p115/Uso1 also contains ARM repeats within its globular head domain and a single CC domain near the C-terminus (38). While the region encompassing the ARM repeats and CC domain is smaller in TGGT1_289120, the *Toxoplasma* protein is considerably larger overall at 2,701 residues while the yeast and human Uso1 proteins contain 1,790 and 962 residues, respectively. Thus, while part of TGGT1_289120 is structurally similar to p115/Uso1, it is unclear whether or not the proteins are direct homologs. While this study was being prepared for publication, a study of the *T. gondii* COG complex by Marsilia et al. independently identified TGGT1_289120 and named it Uso1-like protein 1 (TgULP1) based on its structural similarity to the p115/Uso1 protein (41). To maintain consistency, we adopted the same nomenclature and also named the protein ULP1.

The similarity to the essential vesicular trafficking factor p115/Uso1 and its negative phenotype score of −3.8 in a genome-wide CRISPR/Cas9 screen (GWCS) led us to hypothesize that this protein may play a key role in secretory trafficking in *T. gondii* (42). To determine the localization of ULP1, we used CRISPR/Cas9 to fuse sequences encoding a C-terminal 3xHA epitope tag at its endogenous locus (43, 44). Immunofluorescence assay (IFA) revealed that ULP1 localized to a distinct bar-shaped region anterior to the nucleus, suggesting localization to the Golgi apparatus. To confirm this, we co-stained for GRASP55-YFP, a marker of the cis-medial Golgi apparatus in *T. gondii* and found that the two proteins colocalized (Fig. 1B) (45).

To study the function of ULP1, we used the auxin-inducible degron (AID) system, which allows rapid proteasomal degradation of a target protein upon treatment with indoleacetic acid (IAA) (46, 47). To create an ULP1^AID^ parasite strain, we fused sequences encoding an mAID-3xHA tag to the 3′ end of the gene in a strain that carries the TIR1 auxin-receptor FBOX protein. IFA showed that the degron-tagged protein localizes appropriately and is completely depleted after 24 hours of IAA treatment, which was also confirmed by western blot (Fig. 1C; Fig. S1A). The ULP1-depleted parasites did not appear to exhibit defects in overall morphology. To assess how loss of ULP1 would affect the parasite’s overall lytic ability, we performed plaque assays, which showed a dramatic 88.7% decrease in plaque size when ULP1 was depleted (Fig. 1D and E). Given the low phenotype score (−3.8), we wondered whether the ability to form small plaques may be due to some residual ULP1, which escaped degradation. To determine if this was the case, we used CRISPR/Cas9 to disrupt the endogenous locus for ULP1. Disruption of *ULP1* was confirmed by PCR, and loss of the protein was verified by both IFA and western blot (Fig. 1F and G; Fig. S1B). Using this method, we successfully generated a Δ*ulp1* strain, which phenocopied the plaque defect exhibited by IAA-treated ULP1^AID^ parasites (Fig. 1H and I). To avoid potential issues with functional compensation, which has been previously documented for knockout strains in *T. gondii*, we decided to proceed with the ULP1^AID^ strain for further phenotypic analysis (48–50).

To determine which part of the lytic cycle is disrupted upon ULP1 depletion, we performed assays to assess replication, invasion, and egress using ULP1^AID^ parasites treated with IAA or a vehicle control. At 30 hours post-infection, ULP1-depleted parasites were found primarily in 8-parasite vacuoles, whereas untreated parasites were primarily found in 16-parasite vacuoles (Fig. 1J). Similarly, at 36 hours post-infection, ULP1-depleted parasites were primarily in 8- and 16-parasite vacuoles, while untreated parasites had progressed to mainly 16- and 32-parasite vacuoles (Fig. 1K). Overall, ULP1-depleted parasites were approximately one replication cycle behind untreated parasites at both timepoints. In addition, invasion and egress were reduced in ULP1-depleted parasites by 29% and 41%, respectively (Fig. 1L and M). Together, this demonstrates that ULP1 plays important roles throughout all three stages of the lytic cycle.

**Fig 2:**
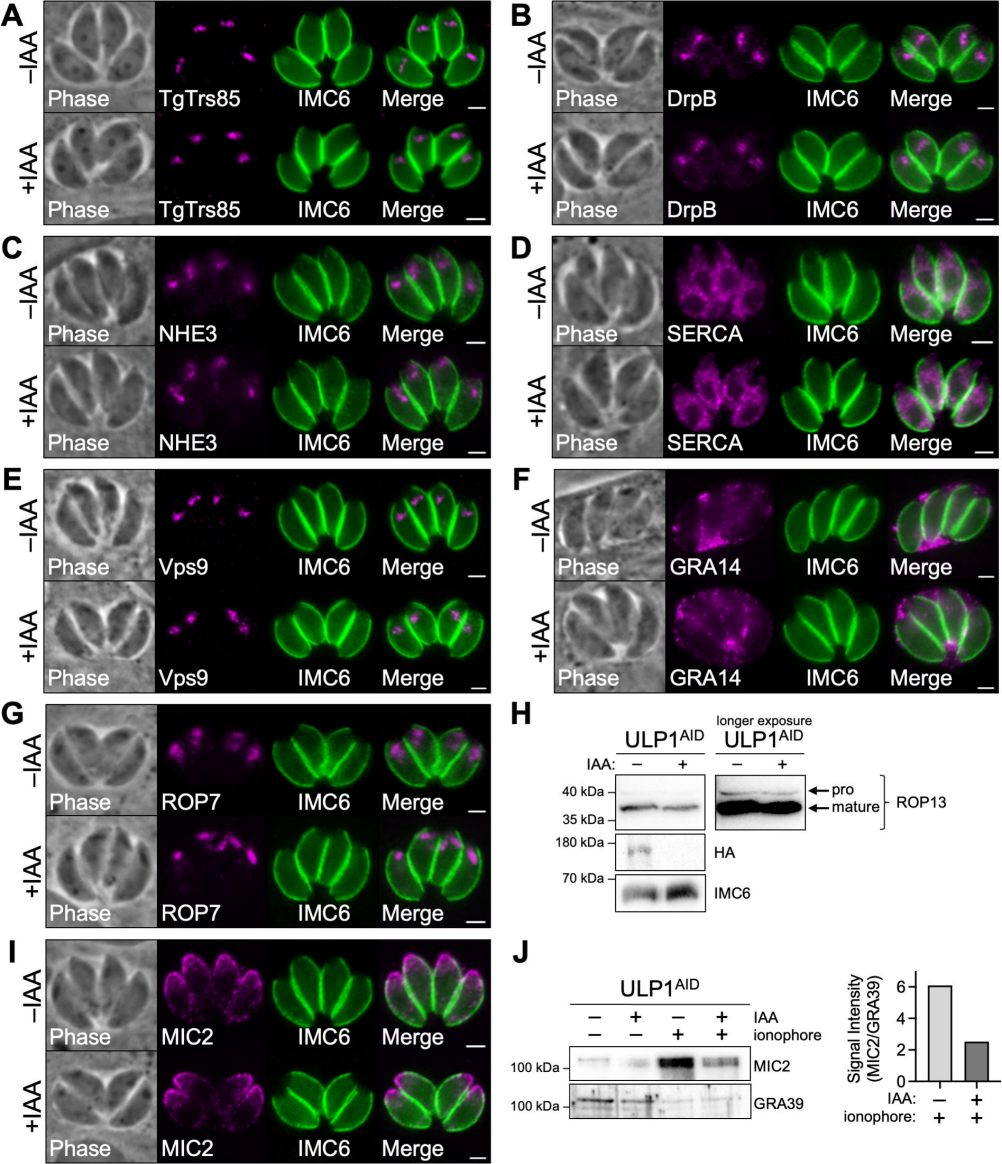
Depletion of ULP1 causes a defect in microneme secretion despite normal organellar morphology and trafficking to secretory organelles. IFAs comparing the overall morphology of various *T. gondii* organelles in control vs. ULP1-depleted parasites. (**A**) Golgi apparatus morphology is unaffected by depletion of ULP1. Magenta, anti-V5 detecting TgTrs85^3xV5^; green, anti-IMC6. (**B**) Post-Golgi vesicle morphology is unaffected by depletion of ULP1. Magenta, anti-DrpB; green, anti-IMC6. (**C**) PLVAC morphology is unaffected by depletion of ULP1. Magenta, anti-NHE3; green, anti-IMC6. (**D**) ER morphology is unaffected by depletion of ULP1. Magenta, anti-SERCA; green, anti-IMC6. (**E**) ELC morphology is unaffected by depletion of ULP1. Magenta, anti-V5 detecting Vps9^3xV5^; green, anti-IMC6. (**F**) Dense granule morphology is unaffected by depletion of ULP1. Magenta, anti-GRA12; green, anti-IMC6. (**G**) Rhoptry morphology is unaffected by depletion of ULP1. Magenta, anti-ROP7; green, anti-IMC6. (**H**) Western blot showing that proROP13 is processed into its mature form normally in ULP1-depleted parasites. Parasites were treated with IAA or vehicle control for 30 hours. IMC6 is used as a loading control. (**I**) Microneme morphology is unaffected by depletion of ULP1. Magenta, anti-MIC2; green, anti-IMC6. (**J**) Western blot and quantification of ULP1^AID^ parasites treated with the calcium ionophore A23187 to induce microneme secretion in the presence and absence of IAA. MIC2 was used to assess microneme secretion, and the constitutively secreted dense granule protein GRA39 was used as a control. Scale bars = 2 µm.

Next, we assessed whether depletion of ULP1 had any effect on various organelles. We began by endogenously tagging the known Golgi protein TgTrs85 to determine whether loss of ULP1 would affect the Golgi apparatus (19). When we depleted ULP1, we found that TgTrs85 was unaffected (Fig. 2A). We next stained for DrpB, a dynamin-related protein that localizes to post-Golgi vesicles, which also appeared to be unaffected (Fig. 2B) (24). Other components of the secretory pathway such as the PLVAC (marked by NHE3), endoplasmic reticulum (SERCA), and ELC (Vps9) were similarly unaffected (Fig. 2C through E). Next, we wanted to determine whether the downstream secretory organelles exhibited altered morphology or protein localization. Targeting and secretion of the dense granule protein GRA14 appeared to be unaffected (Fig. 2F). The rhoptry protein ROP7 was also found to target normally, and the overall morphology of the rhoptries appeared normal (Fig. 2G). We also performed western blot analysis to assess rhoptry protein maturation and found that processing of proROP13 into its mature form occurred normally (Fig. 2H). To assess the micronemes, we stained for MIC2, which targeted properly the micronemes (Fig. 2I). Since we previously observed defects in both invasion and egress, which are both regulated by the release of microneme proteins, we performed a microneme secretion assay. This demonstrated that microneme secretion was reduced by 59% in ULP1-depleted parasites (Fig. 2J). Finally, we stained for the apicoplast (Atrx1) and mitochondrion (F1β ATPase), which appeared morphologically normal (Fig. S2). Together, these data indicate that while there are no gross defects in morphology or overall trafficking to these compartments, loss of ULP1 leads to a reduction in microneme secretion.

### ULP1 proximity labeling and immunoprecipitation yield candidate Golgi proteins

**Fig 3:**
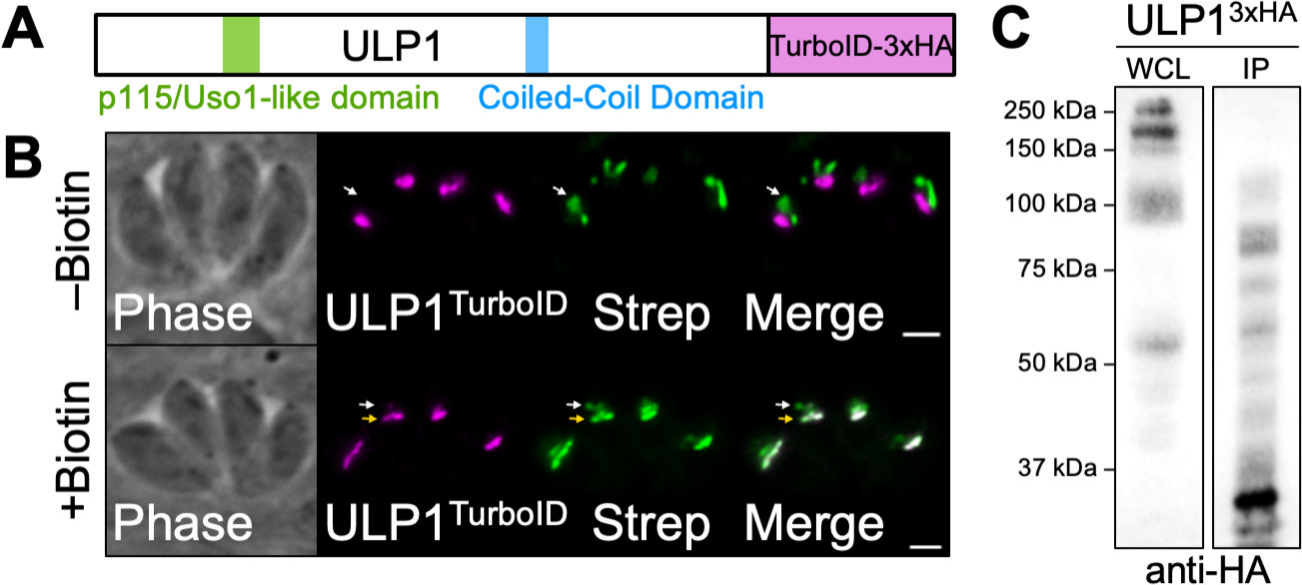
Validation of ULP1 TurboID and IP. (**A**) The TurboID biotin ligase and a 3xHA tag were fused to the C-terminus of ULP1 to generate the ULP1^TurboID^ line. (**B**) IFA showing that ULP1^TurboID^ localizes as expected and results in biotinylation of proximal proteins in a biotin-dependent manner (yellow arrow). The apicoplast, which contains endogenously biotinylated proteins, is denoted by white arrow. Magenta, anti-HA detecting ULP1^TurboID^; green, streptavidin. Scale bar = 2 µm. (**C**) Western blot showing enrichment of ULP1^3xHA^ after immunoprecipitation with anti-HA resin despite significant protein breakdown. WCL, whole cell lysate prior to IP; IP, final sample eluted from anti-HA resin.

To identify additional Golgi proteins, we decided to use ULP1 as bait for both proximity labeling and immunoprecipitation (IP) experiments. For proximity labeling, the biotin ligase TurboID-3xHA was fused to the C-terminus of ULP1 (Fig. 3A) (51). The ULP1^TurboID^ fusion protein was detectable by IFA and trafficked appropriately to the Golgi apparatus. In the absence of biotin, only the endogenously biotinylated apicoplast stained with streptavidin. Upon addition of biotin, the Golgi apparatus also stained with streptavidin, confirming that the ULP1^TurboID^ fusion protein was enzymatically active and biotinylated proximal Golgi proteins (Fig. 3B). The IP analysis was performed on the ULP1^3xHA^ strain. Western blot showed that the 3xHA-tagged protein migrated at a smaller size than expected based on its molecular weight of 290 kDa and exhibited significant protein breakdown. During the process of affinity chromatography for the IP experiment, the protein experienced even more severe breakdown. Despite this, the elution fraction was still highly enriched for the HA-tagged protein (Fig. 3C). Thus, we continued with the experiment.

As expected, the bait protein ULP1 was the top hit identified by mass spectrometry in the IP experiments and was among the top five hits in the TurboID experiment (Tables S1 and S2). DrpB, TgTrs85, TgEpsL, and Vps9, which have all been previously shown to localize to the *Toxoplasma* Golgi apparatus or nearby compartments of the secretory pathway, were highly enriched in our TurboID experiment (Table S1) (19, 24, 52, 53). In addition, many of the top hits are predicted to be involved in processes such as ER-to-Golgi retrograde transport, Golgi organization, and intracellular protein transport. Many of the most enriched proteins were hypothetical proteins that have not previously been studied. Domain analysis allowed us to identify two of these hypothetical proteins, TGGT1_278890 and TGGT1_262450, as homologs of the known Golgi proteins CDP/cut alternatively spliced product (CASP) and dymeclin, respectively.

Our IP experiment identified several interesting candidate interactors, including the hypothetical protein TGGT1_216370, which was also among the top hits in the TurboID experiment (Table S2). In addition, homologs of all eight subunits of the COG complex immunoprecipitated with ULP1 and were highly enriched in our TurboID experiment. All eight are currently annotated as hypothetical proteins, but BLASTp and functional domain analysis support their designation as COG complex subunits, although the BLASTp *e*-value for COG1 is weak (*e*^−6^) (25). As p115/Uso1 is a known interactor of the COG complex via direct binding to COG2, this result suggests that ULP1 may have a similar function (54). Several additional proteins were found to immunoprecipitate with ULP1, although fewer peptides were recovered for these proteins. Among these lower abundance hits were a Sec23/Sec24 trunk domain-containing protein, TgEpsL, COP1 subunit ε, the cyclin-dependent kinase (CDK)-related kinase Crk2, Vps35, Rab7, AP1-γ, and DrpB.

### Verification of candidate Golgi proteins

To continue our study of the *Toxoplasma* Golgi apparatus, we selected 11 uncharacterized proteins with GWCS phenotype scores of less than −2 from our TurboID and IP hits (Table 1). Candidates were prioritized based on functional domain analysis and orthology to known Golgi proteins. Five of the selected genes appeared to be direct orthologs of known Golgi-associated proteins. TGGT1_290310 is the putative COG1 ortholog that we identified in our ULP1 IP. TGGT1_311400 is annotated as WD domain, G-beta repeat-containing protein, but BLASTp analysis revealed its identity as Sec31. TGGT1_232190 contains a Sec7 domain within a larger Golgi Brefeldin A resistance guanine nucleotide exchange Factor 1 (GBF1) domain. TGGT1_264090 contains a TRAPPC11 domain. TGGT1_301410 contains both an ENTH domain and a Tepsin domain. Two proteins contain functional domains that suggested they may have functions related to the secretory system but do not appear to be direct orthologs of any known Golgi proteins. TGGT1_294730 contains an ancestral coatomer element 1 (ACE1) domain from Sec16/Sec31 yet lacked other key domains found in these proteins. TGGT1_230400 contains a WIP-related protein domain but appears to only have orthologs in other coccidian parasites (55). The final four selected genes (TGGT1_216370, TGGT1_207370, TGGT1_258080, and TGGT1_240220) are hypothetical proteins that contained no identifiable functional domains aside from predicted CC domains and had orthologs only in cyst-forming coccidia.

**TABLE 1:** Summary of Golgi-associated proteins identified in this study^*a*^

Gene ID	Abbrv.	Protein name	TurboID		IP		ToxoDB annotation	GWCS
			Ctrl	Exp	Ctrl	Exp		
TGGT1_289120	ULP1	Uso1-Like Protein 1	1.5	341	0	382	Hypothetical protein	−3.8
TGGT1_311400	Sec31	Secretory 31	28	698.5	0	0	WD domain, G-beta repeat-containing protein	−5.53
TGGT1_294730	Sec16-L	Sec16-like Protein	12	468	0	0	Hypothetical protein	−2.21
TGGT1_264090	TRAPPC11	Trafficking protein particle complex subunit 11	2	326.5	0	0	Hypothetical protein	−4.74
TGGT1_290310	COG1	Conserved Oligomeric Golgi complex component 1	0	199	0	59	Hypothetical protein	−3.75
TGGT1_232190	GBF1	Golgi Brefeldin A resistance guanine nucleotide exchange Factor 1	1	157	0	0	Sec7 domain-containing protein	−5.57
TGGT1_207370	GLP2	Golgi Localizing Protein 2	1	126.5	0	0	Hypothetical protein	−3.89
TGGT1_230400	WPDP	WIP-related Protein Domain-containing Protein	2	67	0	0	Hypothetical protein	−2.42
TGGT1_216370	GLP3	Golgi Localizing Protein 3	0	46	0	38	Hypothetical protein	−4.37
TGGT1_301410	Tepsin	AP-4 complex accessory subunit Tepsin	0	41	0	0	Hypothetical protein	−4.16
TGGT1_258080	GLP1	Golgi Localizing Protein 1	0	32.5	0	0	Hypothetical protein	−2.41
TGGT1_240220	GLP4	Golgi Localizing Protein 4	0	20	0	0	Hypothetical protein	−2.1

^*a*^ Table summarizing the 12 Golgi-associated proteins identified and characterized in this study, listed in order of enrichment in the ULP1 TurboID experiment. Average spectral counts for each protein in the TurboID and IP experiments are shown (“Ctrl,” control; “Exp,” experimental). GWCS, phenotype score assigned in a genome-wide CRISPR/Cas9 screen (42); Abbrv., protein name abbreviation.

**Fig 4:**
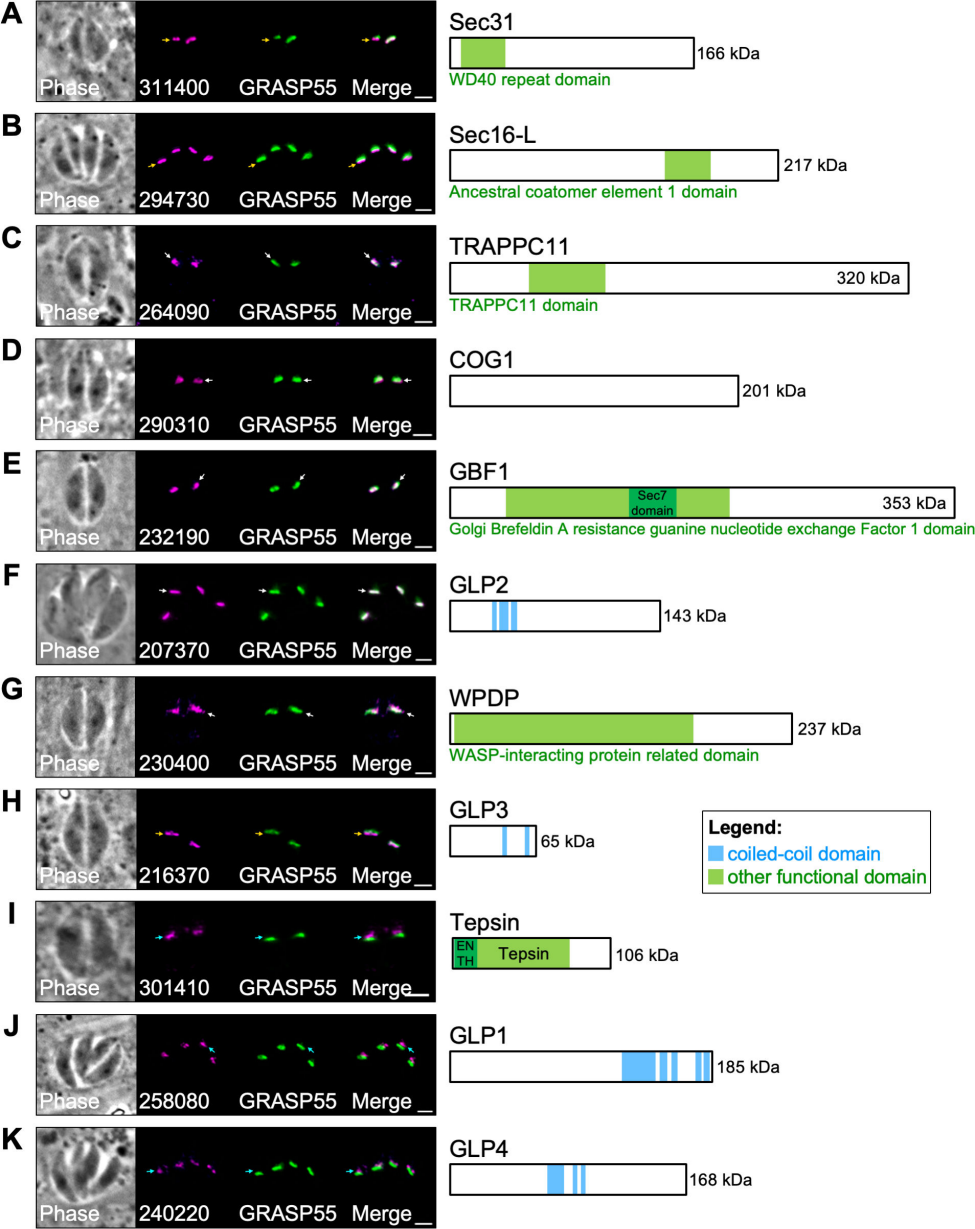
ULP1 TurboID and IP experiments reveal 11 Golgi-associated proteins. IFAs and corresponding gene models for 11 genes identified as candidate Golgi proteins in the ULP1 TurboID and IP experiments. Each gene was endogenously tagged with mAID-3xHA or mIAA7-3xHA AIDs. White arrows indicate that the protein primarily colocalizes with GRASP55-YFP. Yellow arrows indicate that the protein primarily localizes upstream of GRASP55-YFP. Cyan arrows indicate that the protein primarily localizes downstream of GRASP55-YFP. (**A**) TGGT1_311400 (Sec31) localizes upstream of GRASP55 and contains a WD40 repeat domain. (**B**) TGGT1_294730 (Sec16-L) localizes upstream of GRASP55 and contains an ACE1 domain. (**C**) TGGT1_264090 (TRAPPC11) colocalizes with GRASP55 and contains a TRAPPC11 domain. (**D**) TGGT1_290310 (COG1) colocalizes with GRASP55 and contains no identifiable functional domains. (**E**) TGGT1_232190 (GBF1) colocalizes with GRASP55 and contains a GBF1 domain. (**F**) TGGT1_207370 (GLP2) colocalizes with GRASP55 and contains three predicted CC domains within residues 273–301, 313–373, and 382–423. (**G**) TGGT1_230400 (WPDP) colocalizes with GRASP55 and contains a large WASP-interacting protein-related domain. (**H**) TGGT1_216370 (GLP3) localizes downstream of GRASP55 and contains two predicted CC domains within residues 337–370 and 494–527. (**I**) TGGT1_ 301410 (Tepsin) localizes downstream of GRASP55 and contains ENTH and Tepsin domains. (**J**) TGGT1_258080 (GLP1) localizes downstream of GRASP55 and contains five predicted CC domains within residues 1117–1332, 1362–1407, 1425–1464, 1603–1646, and 1659–1701. (**K**) TGGT1_240220 (GLP4) localizes downstream of GRASP55 and contains three predicted CC domains within residues 634–747, 797–830, and 850–881. Magenta, anti-HA detecting degron-tagged proteins; green, GRASP55 YFP. Scale bars = 2 µm.

To verify these candidate Golgi proteins, we endogenously tagged each protein at its C-terminus with an AID coupled to a 3xHA epitope tag in a TIR1 strain (46). Integration of the degron at the correct locus was confirmed by PCR (Fig. S3). We then expressed the Golgi marker GRASP55-YFP in each line and used IFA to assess the localization of each gene (45). All 11 genes either colocalized with GRASP55 or were closely adjacent, confirming their association with the *Toxoplasma* Golgi apparatus (Fig. 4). Based on their localization and functional domains, we assigned names to each of the 11 proteins. TGGT1_311400 was designated Sec31, TGGT1_294730 as Sec16-like protein (Sec16-L), TGGT1_264090 as TRAPPC11-like protein (TRAPPC11), TGGT1_290310 as COG1, TGGT1_232190 as GBF1, and TGGT1_230400 as WIP-related Protein Domain-containing Protein (WPDP). The hypothetical protein TGGT1_258080 was also recently identified by Marsilia et al., so we adopted their nomenclature and named it Golgi Localizing Protein 1 (GLP1). The hypothetical proteins TGGT1_216370, TGGT1_207370, and TGGT1_240220 were designated as Golgi Localizing Proteins 2, 3, and 4, respectively (GLP2-4). Upon further inspection, we noticed that the extent to which each protein colocalized with GRASP55 varied. Sec31, Sec16-L, and GLP3 appeared to localize upstream of GRASP55 in the secretory pathway (Fig. 4A, B and H). Sec31 and Sec16 are both known to localize to ER exit sites where they play roles in anterograde transport (56). While GLP3 does not contain any domains that hint at its function, its similar localization to Sec31 and Sec16-L suggests it may also play a role in early anterograde transport. TRAPPC11, COG1, GBF1, GLP2, and WPDP all appeared to mostly colocalize with GRASP55, indicating a primary localization in the cis-medial Golgi (Fig. 4C through G and I). TRAPPC11 and WPDP displayed some additional signal downstream of GRASP55, suggesting they may also localize to the *trans* Golgi network (Fig. 4C and G). Finally, Tepsin, GLP1, and GLP4 localized downstream of GRASP55, indicating that they likely are restricted to the *trans* Golgi network (Fig. 4J and K).

### Functional analysis of Golgi-associated proteins

**Fig 5:**
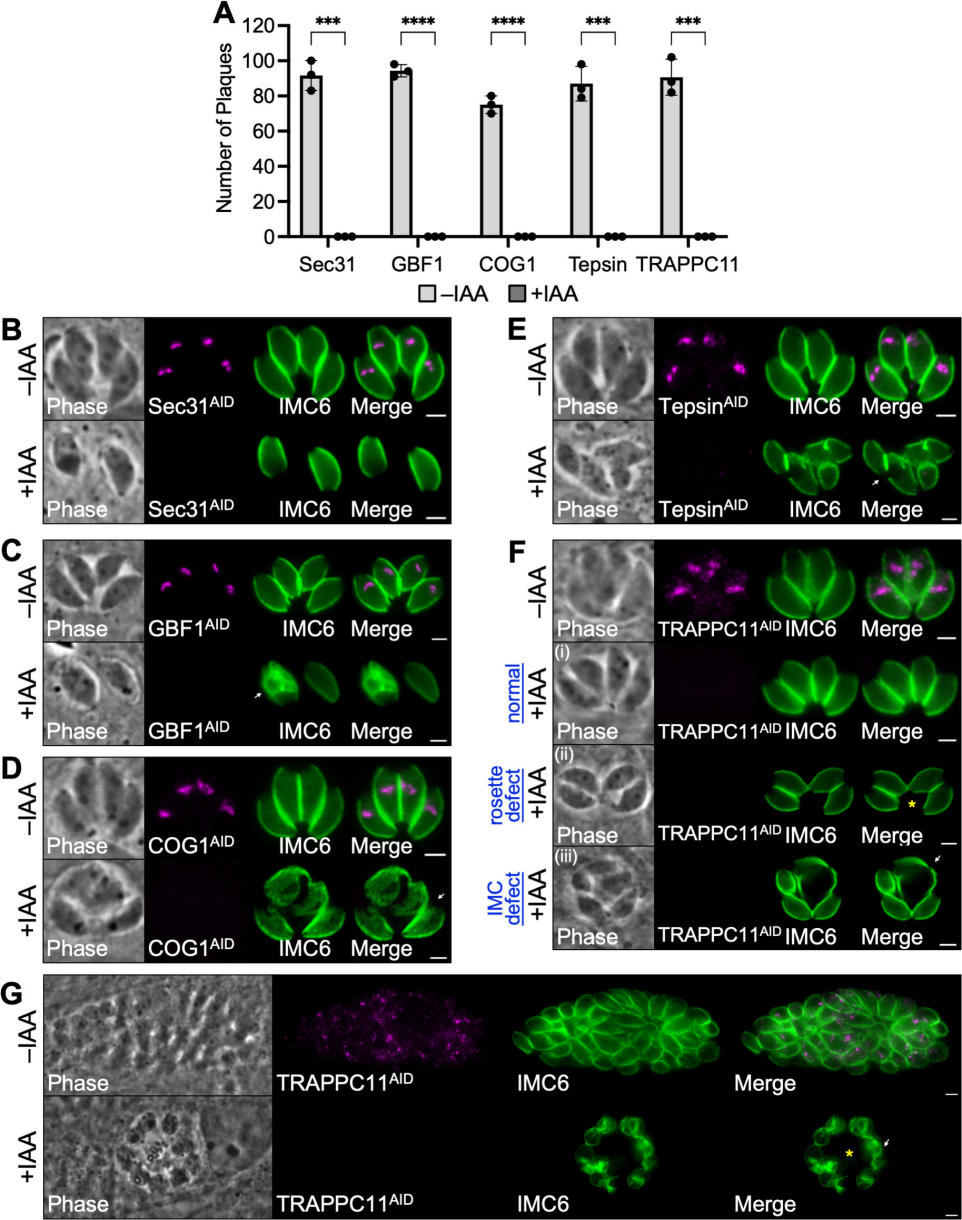
Conditional knockdown of Sec31, GBF1, COG1, Tepsin, and TRAPPC11. (**A**) Plaque assays were performed ±IAA to assess how depletion of Sec31, GBF1, COG1, Tepsin, or TRAPPC11 affects overall lytic ability over the course of 7 days. Number of plaques was quantified for each condition. Statistical significance was determined using multiple two-tailed t tests (****P < 0.0001 and ***P < .001). (**B, C**) Depletion of Sec31 or GBF1 results in growth arrest. Depletion of GBF1 additionally leads to morphological defects in some parasites (white arrow). (**D, E**) Depletion of COG1 or Tepsin results in morphological defects (white arrows). (**F**) Depletion of TRAPPC11 for 24 hours has no obvious effect on most parasites (panel i) but causes some parasites to exhibit abnormal rosette formation (panel ii, yellow asterisk) or morphological defects (panel iii, white arrow). (**G**) Depletion of TRAPPC11 for 43 hours results in severe defects in growth and IMC morphology (white arrow), as well as abnormal vacuolar arrangement (yellow asterisk). Magenta, anti-HA detecting degron-tagged proteins; green, anti-IMC6. Scale bars = 2 µm.

We next used the AID system to assess how depletion of each protein would affect parasite fitness. We began by performing western blot analyses to verify that each degron-tagged protein was efficiently depleted after 24 hours of IAA treatment (Fig. S4). We then performed IFAs and plaque assays to assess the phenotype of each knockdown strain. Five of the 11 strains (Sec31, GBF1, COG1, Tepsin, and TRAPPC11) were found to be completely deficient in plaque formation, indicating that these proteins are essential for parasite survival (Fig. 5A). Sec31 exhibited a severe growth arrest, with nearly all vacuoles containing only a single parasite after 24 hours of growth in the presence of IAA (Fig. 5B). Despite the severe growth defect, Sec31-depleted parasites appeared to have normal overall morphology as assessed by IMC6 staining. GBF1 also exhibited a severe growth arrest (Fig. 5C). However, in contrast to Sec31, many GBF1-depleted parasites also exhibited defective IMC morphology. Both COG1 and Tepsin-depleted parasites did not exhibit growth arrest at 24 hours but had severe defects in morphology, such as large gashes in the cytoskeleton marked by IMC6 (Fig. 5D and E). The last essential protein, TRAPPC11, did not exhibit growth arrest, and most vacuoles appeared morphologically normal after 24 hours of IAA treatment (Fig. 5F, panel i). However, many TRAPPC11-depleted parasites displayed abnormal rosette formation, in which the basal ends of the parasites within a single vacuole were spaced apart farther than usual (Fig. 5F, panel ii). Additionally, a small number of TRAPPC11-depleted parasites displayed defects in IMC morphology (Fig. 5F, panel iii). To determine whether these defects worsened over time, we extended IAA treatment to 43 hours (Fig. 5G). At this timepoint, all TRAPPC11-depleted parasites exhibited severe defects in IMC morphology and growth. Interestingly, we also observed many vacuoles where the parasites were arranged in a ring shape with a large, seemingly empty space in the center of the vacuole.

**Fig 6:**
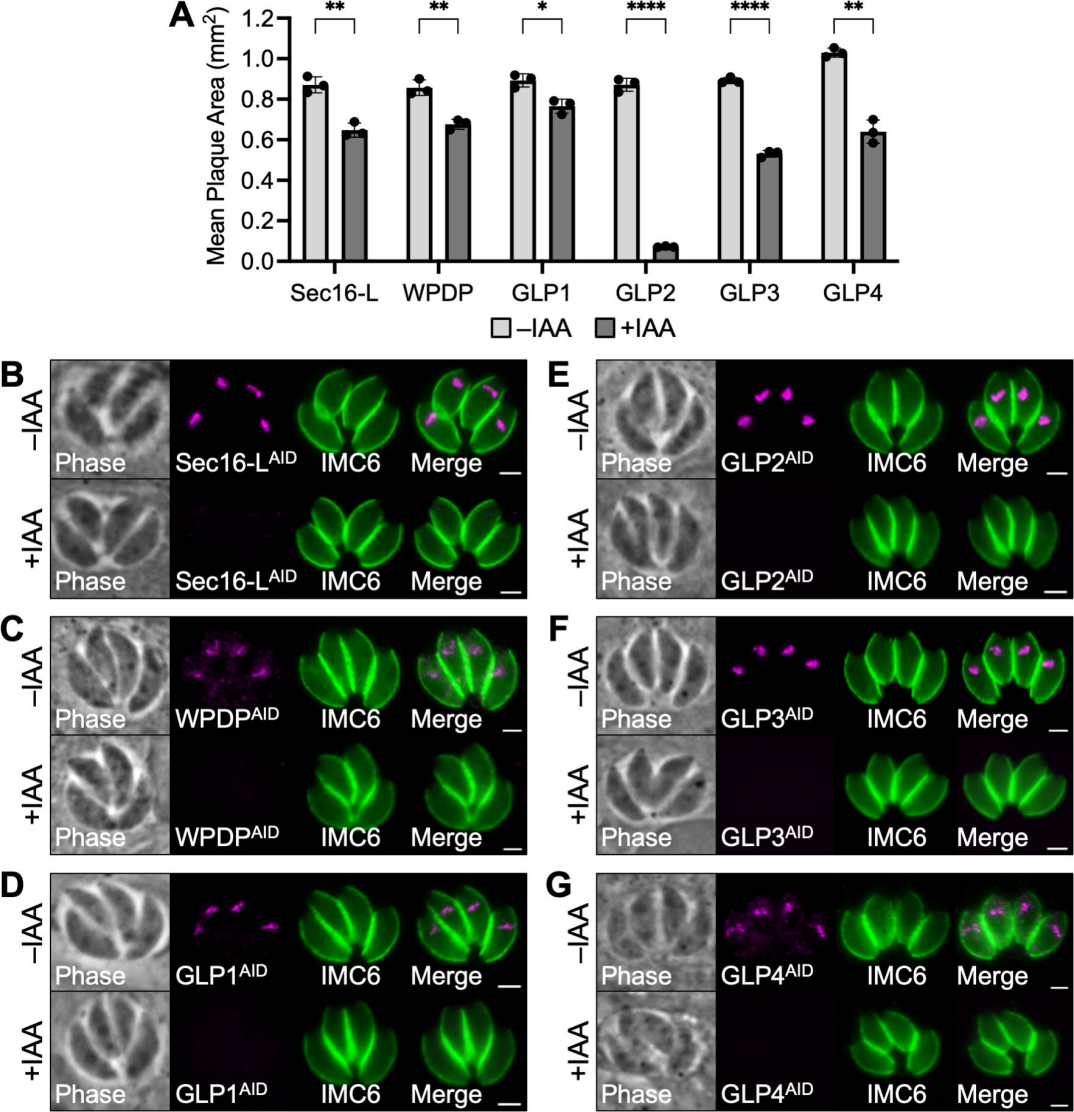
Conditional knockdown of Sec16-L, WPDP, GLP1, GLP2, GLP3, and GLP4. (**A**) Plaque assays were performed ±IAA to assess how depletion of Sec16-L, WPDP, GLP1, GLP2, GLP3, and GLP4 affects overall lytic ability over the course of 7 days. Mean plaque area was quantified for each condition. Statistical significance was determined using multiple two-tailed t tests (****P < 0.0001, ***P < 0.001, **P < 0.01, and *P < 0.05). (**B–G**) Depletion of Sec16-L, WPDP, GLP1, GLP2, GLP3, or GLP4 does not result in any obvious defects in morphology. Magenta, anti-HA detecting degron-tagged proteins; green, anti-IMC6. Scale bars = 2 µm.

The remaining six proteins were found by plaque assay to be non-essential, but their depletion resulted in a statistically significant reduction in lytic ability (Fig. 6A). The severity of the defect varied widely among the six proteins. The most severe growth defect (91.8%) was observed for GLP2. The remaining five proteins exhibited more modest reductions—25.7% for Sec16-L, 21.0% for WPDP, 14.2% for GLP1, 40.5% for GLP3, and 37.9% for GLP4. Despite the reduction in fitness, all six of these proteins exhibited no obvious defects in morphology by IFA (Fig. 6B through G).

## DISCUSSION

In this study, we identified and characterized the Golgi protein ULP1 as a critical regulator of parasite growth and used it as a probe to identify and characterize additional Golgi proteins. Our analysis of ULP1 demonstrated that the protein localizes to the cis-medial Golgi and plays an important role in all phases of the parasite’s lytic cycle. Loss of the protein by either AID-mediated knockdown or gene knockout resulted in a severe defect in plaque size as well a reduction in replication rate, microneme secretion, invasion, and egress. Our findings contrast with that of Marsilia et al. who showed a complete loss of lytic ability upon ULP1 knockdown and place its primary localization in the trans-Golgi network (41). While the conditional knockdown strains may vary in the phenotypes observed, the ULP1 knockout indicates that the protein is not strictly required for parasite survival.

ULP1 is an interesting protein because it is unique to cyst-forming coccidia yet is structurally homologous to the conserved eukaryotic trafficking factor p115/Uso1. Studies in mammalian cells, *Drosophila*, and yeast have demonstrated that p115/Uso1 has a wide range of essential functions including tethering COPI vesicles to the Golgi, transporting vesicles from the ER to the Golgi, and facilitating SNARE complex assembly (29–34). Loss of p115/Uso1 has been shown to result in fragmentation of the Golgi and accumulation of Golgi-derived vesicles (57). While we did not identify any apparent defects in trafficking of markers to key secretory organelles or loss of Golgi integrity upon ULP1 knockdown, we did find a significant reduction in microneme secretion, which led to defects in both invasion and egress. We also observed a significant reduction in replicative ability. While this could be a result of the delay in invasion that we observed, it could also suggest that ULP1 plays a role in cell cycle regulation. In other systems, p115/Uso1 has been found to associate with the spindle poles in addition to the Golgi apparatus, and loss of the protein has been shown to impair mitosis (58, 59). However, since ULP1 does not localize to the spindle poles, it is unlikely to regulate the cell cycle by the same mechanism as p115/Uso1.

P115/Uso1 is known to interact with several trafficking proteins including Rab1, GBF1, COG2, GM130, and SNARE complex components Syntaxin 5, Bet1, and Bos1 (32, 54, 60–62). Our IP analysis was highly enriched for all eight COG complex subunits, with COG2 being the second most enriched. Marsilia et al. also identified all eight components of the COG complex in their ULP1 IP, further supporting the legitimacy of these interactions (41). Interestingly, we did not identify any of the other known p115/Uso1-interacting proteins. While these other proteins may have been lost during processing, it is also possible that ULP1 function has diverged enough from p115/Uso1 such that it does not bind to these proteins and instead uses other parasite-specific proteins to fulfill these roles. Supporting this, the Golgi protein GLP2 was found to undergo IP with ULP1 in both Marsilia et al.’s study and our study. p115/Uso1 has been shown to bind to Cdk1, and loss of p115 was demonstrated to reduce Cdk1 activation (59). Interestingly, we identified a CDK-related kinase, Crk2, in our IP analysis. Given that ULP1 depletion leads to a decrease in replication rate, it is possible that interaction with Crk2 plays a role in *T. gondii* mitosis. Alternatively, the replication defect could be due to general ER stress caused by alteration of the secretory pathway (63). When comparing the remaining results of our IP with those of Marsilia et al.’s, several differences are observed. They identified a wide variety of other proteins including eight SNARE proteins, four AP-5 complex proteins, and five COPI complex subunits that either were absent from our analysis or did not reach the twofold enrichment cut-off. Additionally, the authors noted that over 20 IMC proteins and Rab11b, which is critical for IMC biogenesis, were enriched in their ULP1 IP. However, we did not observe enrichment for any of these proteins. Similarly, our IP identified Crk2, Rab7, Vps35, TgEpsL, AP1-γ, and a Sec23/Sec24 trunk domain-containing protein while the other study did not. These disparities likely arise from differences in sample processing. Thus, further analysis of ULP1 candidate binding partners will be needed to create a more definitive picture of the ULP1 interactome. Since IP analysis does not distinguish between direct and indirect interactions, it would be interesting to determine direct interactors using yeast two-hybrid analyses or similar approaches.

Using ULP1 as bait in both TurboID and IP experiments, we were able to identify 11 previously uncharacterized Golgi-associated proteins. Sec31, COG1, GBF1, Tepsin, and TRAPPC11 were found to be essential, which is unsurprising due to their critical conserved functions in other eukaryotes. Two of these proteins, Sec31 and GBF1, caused an immediate growth arrest upon depletion, likely due to a block in the secretory pathway that prevents the continuation of the cell cycle. Sec31 acts as a component of the COPII complex, which facilitates anterograde transport from the ER to the Golgi and causes growth arrest when depleted in *Trypanosomes* (56, 64, 65). We observed the *T. gondii* Sec31 localizing upstream of GRASP55 in the secretory pathway, indicating that it likely localizes to ER exit sites as in other eukaryotes. The localization of this protein to this subcompartment of the Golgi represents a valuable new marker for this region of the secretory pathway in *T. gondii*. GBF1 is a Golgi-resident guanine nucleotide exchange factor that regulates COPI vesicle-mediated transport by activating ADP-ribosylation factor proteins (66, 67). We observed *Toxoplasma* GBF1 localizing to the cis-medial Golgi, which agrees with its function in other systems.

Unlike Sec31 and GBF1, the remaining three essential proteins, COG1, Tepsin, and TRAPPC11, did not exhibit an immediate growth arrest upon depletion. COG1 is a component of the COG complex, which acts in the retrograde transport pathway by tethering COPI-coated vesicles that are used for recycling Golgi-resident enzymes such as glycosyltransferases back to the Golgi (27, 28). Several *T. gondii* proteins such as GAP50 and CST1 have been shown to be glycosylated, and this modification is required for their proper targeting and function (68, 69). In agreement with this, Marsilia et al. demonstrated that O-glycosylation is disrupted upon COG3 or COG7 depletion (41). This function is primarily carried out in the *cis*/medial Golgi, which matches our observed colocalization of COG1 with GRASP55. Tepsin is an accessory protein that associates with the AP-4 complex, which plays a role in vesicular trafficking of proteins at the trans-Golgi network (70, 71). Knockdown of COG1, Tepsin, or TRAPPC11 resulted in severe defects in IMC morphology, suggesting that loss of these proteins causes issues with the secretory pathway ultimately leading to defects in IMC biogenesis. Interestingly, depletion of COG1 or Tepsin led to morphological defects much earlier than depletion of TRAPPC11. In addition, depletion of TRAPPC11 caused an interesting defect in vacuolar arrangement in which parasites appeared to be pushed toward the edge of the vacuole, leaving a large, seemingly empty space in the center. The source of this defect is unclear, but ultrastructural studies may provide more insight.

Two of the identified proteins, Sec16-L and WPDP, do not appear to be direct orthologs of known Golgi proteins but contain domains that suggested conserved functions. Sec16-L contains the ACE1 domain from Sec16, an essential COPII vesicle coat protein required for ER transport vesicle budding and formation of ER exit sites (72). However, it is missing the Sec16-conserved C-terminal domain, which facilitates binding to Sec23 and is essential for function (73). Sec16-L’s localization upstream of GRASP55 further supports its functional similarity to Sec16 and provides another marker of this early subcompartment of the secretory pathway in addition to Sec31. While Sec16 is essential in other systems, Sec16-L had a relatively modest fitness defect upon conditional knockdown and its ortholog in *P. falciparum* (PF3D7_1119900) is also predicted to be dispensable (74). It is possible that another protein has adopted this function in *T. gondii* and other apicomplexans, rendering this protein redundant. WPDP contains a WIP-related protein domain, which is known to facilitate actin binding. Numerous Golgi-resident actin-binding proteins have previously been identified in mammalian cells, and the actin cytoskeleton has been shown to be critical for maintaining Golgi structure and facilitating vesicular trafficking in both mammalian cells and *T. gondii* (75, 76). Similar to Sec16-L, WPDP had a modest phenotype upon conditional knockdown. It is possible that WPDP may be one of several actin-binding proteins that help maintain Golgi structure in *T. gondii* and related apicomplexans.

We additionally identified four parasite-specific Golgi proteins, GLP1-4, which were all found to be non-essential. GLP3, which we identified as an interactor of ULP1 by IP, was found to localize upstream of GRASP55 like Sec16-L and Sec31 and therefore could play a role in ER to Golgi vesicle transport. GLP1 and GLP4 both localize downstream of GRASP55, suggesting they function in the trans-Golgi network. Both proteins exhibit only a relatively small reduction in plaque size upon their depletion. Interestingly, the two proteins are similar in size and both contain a region with one larger CC domain closely followed by two shorter CC domains. While their sequence similarity does not suggest they are paralogs, their similar localizations, phenotypes, and structural compositions could indicate possible functional redundancy. Finally, GLP2 was found to localize to the cis-medial Golgi and caused a dramatic 91.8% reduction in plaque size when depleted. Despite this, we did not observe any obvious growth defects or morphological changes by IFA, suggesting that loss of GLP2 may affect either invasion or egress rather than replication. Interestingly, all of the GLPs contain several predicted CC domains and are only found in the cyst-forming coccidia. CC domain-containing proteins such as the golgin proteins GM130, giantin, and CASP are known to act as tethering factors in other eukaryotes (77). As the GLPs are unique to the cyst-forming coccidia, it is possible they could use their CC domains to act as Golgi tethering factors that carry out unique functions in this subset of apicomplexan parasites.

Together, this study reveals a total of 12 Golgi-associated proteins including several that are essential and others that are parasite specific. Their identification provides valuable new markers for the secretory pathway and provides exciting candidates for further functional analysis. Due to the apicomplexan-specific nature of several of these proteins, they represent both novel cell biology and potential targets for therapeutic intervention.

## MATERIALS AND METHODS

### *T. gondii* and host cell culture

Parental *T. gondii* RHΔ*hxgprtΔku80* (wild-type) and subsequent strains were grown on confluent monolayers of human foreskin fibroblasts (BJ, ATCC, Manassas, VA) at 37°C and 5% CO_2_ in Dulbecco’s modified Eagle medium (DMEM) supplemented with 5% fetal bovine serum (Gibco), 5% Cosmic calf serum (HyClone), and 1× penicillin-streptomycin-L-glutamine (Gibco). Constructs containing selectable markers were selected using 1 µM pyrimethamine (dihydrofolate reductase-thymidylate synthase ), 50 µg/mL mycophenolic acid-xanthine (HXGPRT), or 40 µM chloramphenicol (CAT) (78–80). For AID conditional knockdown experiments, media were supplemented with 500 µM indoleacetic acid (Sigma-Aldrich; I2886) or vehicle control.

### Antibodies

The HA epitope was detected with mouse monoclonal antibody (mAb) HA.11 (BioLegend; 901515). The V5 epitope was detected with mouse mAb anti-V5 (Invitrogen; R96025). *Toxoplasma*-specific antibodies include rabbit polyclonal (pAb) anti-IMC6 (81), rat pAb anti-DrpB (24), guinea pig pAb anti-NHE3 (82), mouse pAb anti-SERCA (83), mouse mAb anti-ROP7 (1B10) (84), mouse mAb anti-MIC2 (6D10) (85), mouse pAb anti-GRA14 (84), mouse mAb anti-ATrx1 (11G8) (86), mouse mAb anti-F1β subunit (5F4) (87), mouse pAb anti-ROP13 (88), rabbit pAb anti-Catalase (89), mouse pAb anti-SAG1 (90), and rat pAb anti-GRA39 (91).

### Endogenous epitope tagging and knockout

For C-terminal endogenous tagging, a pU6-Universal plasmid containing a protospacer against the 3′ untranslated region (UTR) of the target protein approximately 100 bp downstream of the stop codon was generated, as described previously (43). A homology-directed repair (HDR) template was PCR amplified using the Δ*ku80*-dependent LIC vector pmAID3xHA.LIC-HPT, pmIAA73xHA.LIC-HPT, p3xHA.LIC-DHFR, p3xV5.LIC-DHFR, or pTurboID3xHA.LIC-DHFR, all of which include the epitope tag, 3′ UTR, and a selection cassette (92). The 60-bp primers include 40 bp of homology immediately upstream of the stop codon or 40 bp of homology within the 3′ UTR downstream of the CRISPR/Cas9 cut site. For knockout of ULP1, the protospacer was designed to target the coding region of ULP1, ligated into the pU6-Universal plasmid, and prepared similarly to the endogenous tagging constructs. The HDR template was PCR ampliﬁed from a pJET vector containing the HXGPRT drug marker driven by the NcGRA7 promoter using primers that included 40 bp of homology immediately upstream of the start codon or 40 bp of homology downstream of the region used for homologous recombination for endogenous tagging. All primers that were used for pU6-Universal plasmids and HDR templates are listed in Table S3.

For all tagging and knockout constructs, approximately 50 µg of the sequence-verified pU6-Universal plasmid was precipitated in ethanol, and the PCR-amplified HDR template was purified by phenol chloroform extraction and precipitated in ethanol. Both constructs were electroporated into the appropriate parasite strain. Transfected cells were allowed to invade a confluent monolayer of HFFs overnight, and appropriate selection was subsequently applied. Successful tagging was confirmed by IFA, and clonal lines of tagged parasites were obtained through limiting dilution.

### Immunofluorescence assay

Confluent HFF cells were grown on glass coverslips and infected with *T. gondii*. After 24–43 hours, the coverslips were fixed with 3.7% formaldehyde in PBS and processed for immunofluorescence as described (88). Primary antibodies were detected by species-specific secondary antibodies conjugated to Alexa Fluor 594/488 (Thermo Fisher). Coverslips were mounted in Vectashield (Vector Labs), viewed with an Axio Imager.Z1 fluorescent microscope, and processed with ZEN 2.3 software (Zeiss).

### Western blot

Parasites were lysed in 1× Laemmli sample buffer with 100 mM DTT and boiled at 100°C for 5 minutes. Lysates were resolved by SDS-PAGE and transferred to nitrocellulose membranes, and proteins were detected with the appropriate primary antibody and corresponding secondary antibody conjugated to horseradish peroxidase. Chemiluminescence was induced using the SuperSignal West Pico substrate (Pierce) and imaged on a ChemiDoc XRS+ (Bio-Rad). For quantification of microneme secretion assays, signal intensity was quantified using ImageLab. The adjusted volume of the MIC2 band relative to the adjusted volume of the corresponding GRA39 loading control band was plotted. Raw data for western blot quantification are shown in Table S4.

### Plaque assay

HFF monolayers were infected with 200 parasites per well of individual strains (±IAA for AID strains) and allowed to form plaques for 7 days. Cells were then fixed with ice-cold methanol and stained with crystal violet. To quantify the plaque size, the areas of 30–50 plaques per condition were measured using ZEN software (Zeiss). To quantify the plaque number, the total number of plaques in each condition was counted manually. All plaque assays were performed in triplicate. Graphical and statistical analyses were performed using Prism GraphPad 8.0. The raw data for all plaque assays can be found in Table S4.

### Quantification of parasites per vacuole

Parasites were pre-treated with IAA or vehicle control for 18 hours prior to infecting coverslips. After coverslips were infected, parasites were allowed to grow ±IAA for 30 or 36 hours prior to fixation and staining with mouse anti-HA (detecting ULP1^AID^) and rabbit anti-IMC6. For each condition, the number of parasites per vacuole (1, 2, 4, 8, 16, or ≥32) was counted for >100 vacuoles across 10 different fields. Experiments were performed in triplicate. Significance was determined using multiple two-tailed t tests.

### Invasion assay

Invasion assays were performed as previously described (93). Parasites were grown for 36 hours ±IAA. Intracellular parasites were harvested and manually lysed using a 27-gauge needle. Parasites were resuspended in Endo buffer (94) and allowed to settle onto coverslips with a monolayer of confluent HFFs for 20 minutes. Endo buffer was replaced with warm D1 media (DMEM, 20 mM HEPES, 1% fetal bovine serum) and incubated at 37°C for 30 minutes. Coverslips were fixed, blocked, and stained with SAG1 to label extracellular parasites. Coverslips were then permeabilized and stained with IMC6 to label all parasites. Parasites were manually scored as invaded (SAG1−, IMC6+) or not invaded (SAG1+, IMC6+) by fluorescence microscopy. Assays were performed in triplicate. At least 200 parasites across at least 10 fields were scored for each replicate.

### Egress assay

Parasites were grown in a monolayer of HFFs on a coverslip for 36 hours ±IAA until most vacuoles contained 16 or 32 parasites. Coverslips were washed twice with warm PBS and then incubated with 1 µm A23187 or DMSO control diluted in PBS at 37°C for 2 minutes. Coverslips were then fixed and stained with IMC12. Vacuoles were manually scored as egressed or not egressed by fluorescence microscopy. Assays were performed in triplicate. At least 100 vacuoles across at least 15 fields were scored for each replicate.

### Microneme secretion assay

Microneme secretion assays were performed as previously described (95, 96). Briefly, parasites were grown for 40 hours ±IAA, and intracellular parasites were collected by mechanical release through a 27-gauge needle. After washing twice in D1 media, parasites were resuspended in prewarmed D1 media containing 1 µM A23187 or DMSO control for 10 minutes at 37°C. Secretion was arrested by cooling on ice, and parasites were pelleted at 1,000 × *g* for 5 minutes at 4°C. The supernatant was collected and centrifuged again at 1,000 × *g*. The resulting supernatant was mixed with 4× sample buffer and assessed by SDS-PAGE and western blot analysis.

### Affinity capture of biotinylated proteins

For affinity capture of proteins from whole cell lysates, HFF monolayers infected with ULP1^TurboID^ or control parasites (RHΔ*hxgprt*Δ*ku80*, WT) were grown in medium containing 150 µM biotin for 30 hours. Intracellular parasites in large vacuoles were collected by manual scraping, washed in PBS, and lysed in radioimmunoprecipitation assay (RIPA) buffer [50 mM Tris (pH 7.5), 150 mM NaCl, 0.1% SDS, 0.5% sodium deoxycholate, and 1% NP-40] supplemented with Complete Protease Inhibitor Cocktail (Roche) for 30 minutes on ice. Lysates were centrifuged for 15 minutes at 14,000 × *g* to pellet insoluble material, and the supernatant was incubated with Streptavidin Plus UltraLink resin (Pierce) overnight at 4°C under gentle agitation. Beads were collected and washed five times in RIPA buffer, followed by three washes in 8M urea buffer [50 mM Tris-HCl (pH 7.4), 150 mM NaCl] (97). Samples were submitted for on-bead digests and subsequently analyzed by mass spectrometry. The experiment was performed in duplicate.

### Immunoprecipitation assay

ULP1^3XHA^ was isolated from 5 × 10^9^ *T. gondii* RH tachyzoites lysed in NP-40 lysis buffer [1% NP-40, 150 mM NaCl, and 50 mM Tris (pH 8.0)] supplemented with complete protease inhibitor (Roche). The insoluble material was removed from the lysate by centrifugation at 10,000 × *g* for 20 minutes. The soluble lysate fraction was incubated with rat anti-HA affinity matrix (Roche) for 3 hours at room temperature with gentle agitation. After washing in NP-40 lysis buffer, the bound protein was eluted using high pH (100 mM triethylamine, pH 11.5), dried to a pellet, and submitted for mass spectrometry.

### Mass spectrometry

Samples were resuspended in digestion buffer [8M urea, 0.1M Tris-HCl (pH 8.5)] and then were reduced, alkylated, and digested by sequential addition of Lys-C and trypsin proteases. Samples were then desalted using C18 tips (Pierce) and fractionated online using a 75-µm inner-diameter fritted fused silica capillary column with a 5-µm pulled electrospray tip and packed in-house with 25 cm of C18 (Dr. Maisch GmbH) 1.9 µm reversed-phase particles. The gradient was delivered by a 140-minute gradient of increasing acetonitrile and eluted directly into a Thermo Orbitrap Fusion Lumos instrument where MS/MS spectra were acquired by Data Dependent Acquisition. Data analysis was performed using ProLuCID and DTASelect2 implemented in Integrated Proteomics Pipeline IP2 (Integrated Proteomics Applications) (98–100). Database searching was performed using a FASTA protein database containing *T. gondii* GT1-translated open-reading frames downloaded from ToxoDB. Protein and peptide identifications were filtered using DTASelect and required a minimum of two unique peptides per protein and a peptide-level false positive rate of less than 5% as estimated by a decoy database strategy. Candidates were ranked by spectral count comparing experimental versus control samples (101). TurboID results were filtered to include only proteins that were at least twofold enriched with a difference of >5 spectral counts when comparing ULP1^TurboID^ samples versus control samples. IP results were filtered to include only proteins that were at least twofold enriched when comparing ULP1^3xHA^ IP samples versus control samples.

### Bioinformatic analysis

Functional domains were identified using a combination of Phyre2, Interpro Domains, PFAM, and Panther analysis (37, 39, 102, 103). Coiled coil domains were predicted using DeepCoil2 using a probability cut-off of 0.5 (40). Orthology was determined using OrthoMCL and BLASTp search against the VEuPathDB database (55).

## SUPPLEMENTAL MATERIAL

Fig. S1 mbio.02380-24-s0001.tiff Western blot validation for ULP1AID and Δulp1 strains.

Fig. S2 mbio.02380-24-s0002.tiff The apicoplast and mitochondrion are unaffected by ULP1 depletion.

Fig. S3 mbio.02380-24-s0003.tiff PCR verification for all Golgi-associated proteins.

Fig. S4 mbio.02380-24-s0004.tiff Western blot validation for Golgi-associated proteins.

Supplemental Legends mbio.02380-24-s0005.docx Legends for supplemental figures and tables.

Table S1 mbio.02380-24-s0006.xlsx ULP1 TurboID results.

Table S2 mbio.02380-24-s0007.xlsx ULP1 immunoprecipitation results.

Table S3 mbio.02380-24-s0008.xlsx Oligonucleotides used in this study.

Table S4 mbio.02380-24-s0009.xlsx Raw data for quantification of plaque assays and western blot.

ASM does not own the copyrights to Supplemental Material that may be linked to, or accessed through, an article. The authors have granted ASM a non-exclusive, world-wide license to publish the Supplemental Material files. Please contact the corresponding author directly for reuse.
