# Supplementary figures and images for: Characterization and functional analysis of Toxoplasma Golgi-associated proteins identified by proximity labeling
Source: mBio. 2024 Sep 30;15(11):e02380-24. doi: 10.1128/mbio.02380-24 (PMC11559087; doi:10.1128/mbio.02380-24)

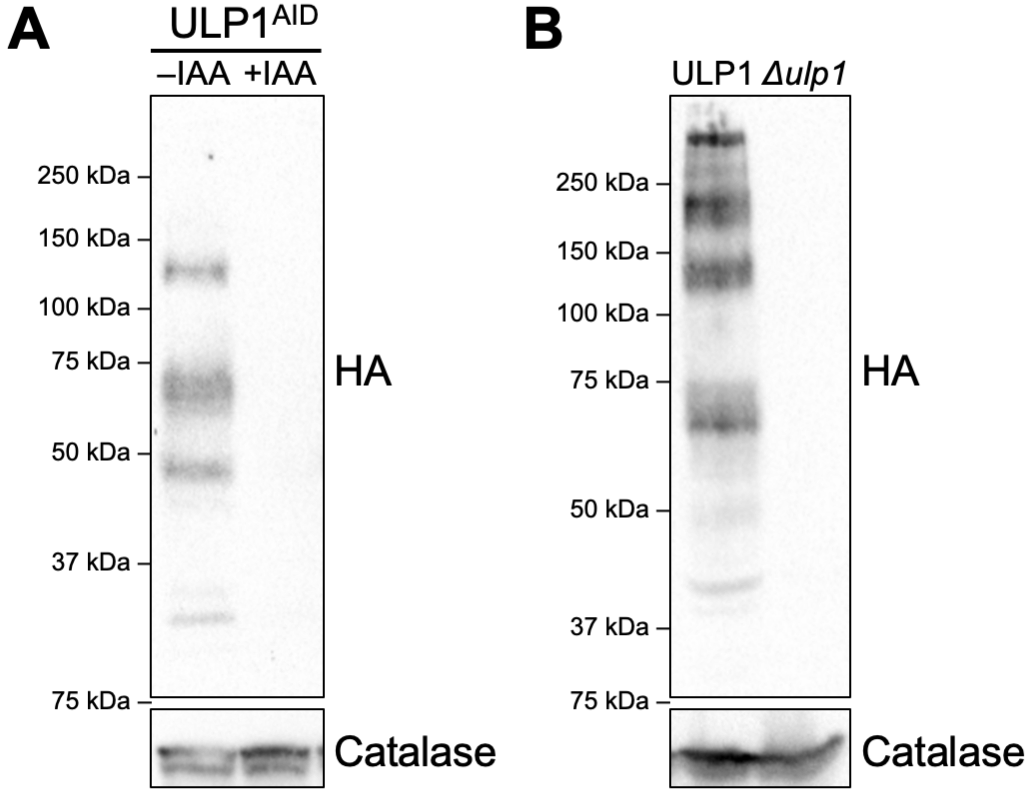

Supplement: Fig. S1 — Western blot validation for ULP1AID and Δulp1 strains. [file mbio.02380-24-s0001.tiff]

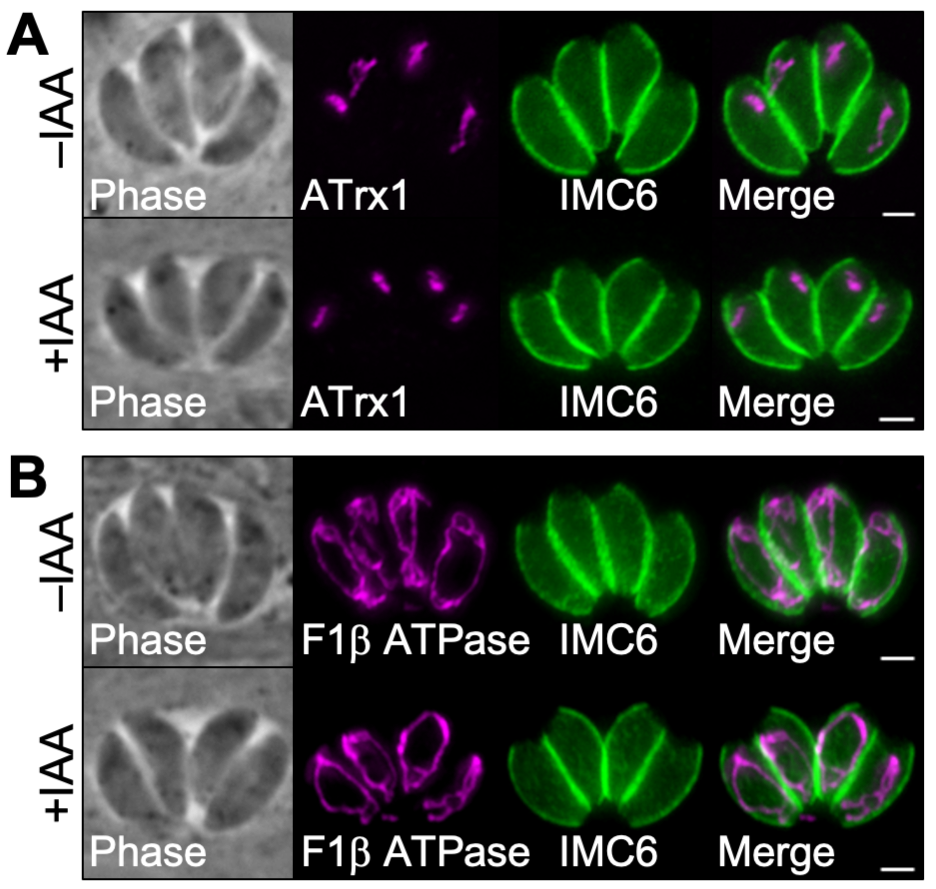

Supplement: Fig. S2 — The apicoplast and mitochondrion are unaffected by ULP1 depletion. [file mbio.02380-24-s0002.tiff]

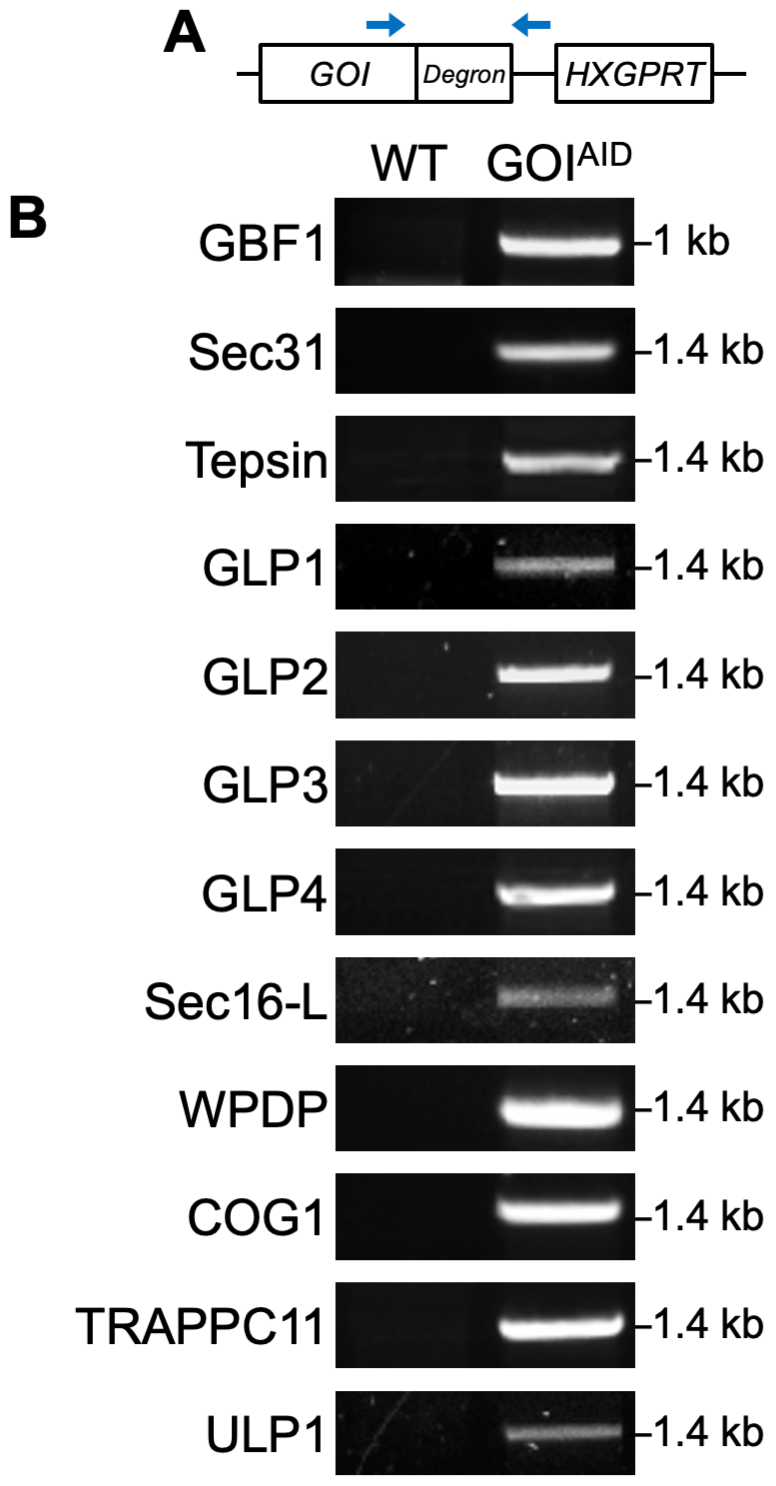

Supplement: Fig. S3 — PCR verification for all Golgi-associated proteins. [file mbio.02380-24-s0003.tiff]

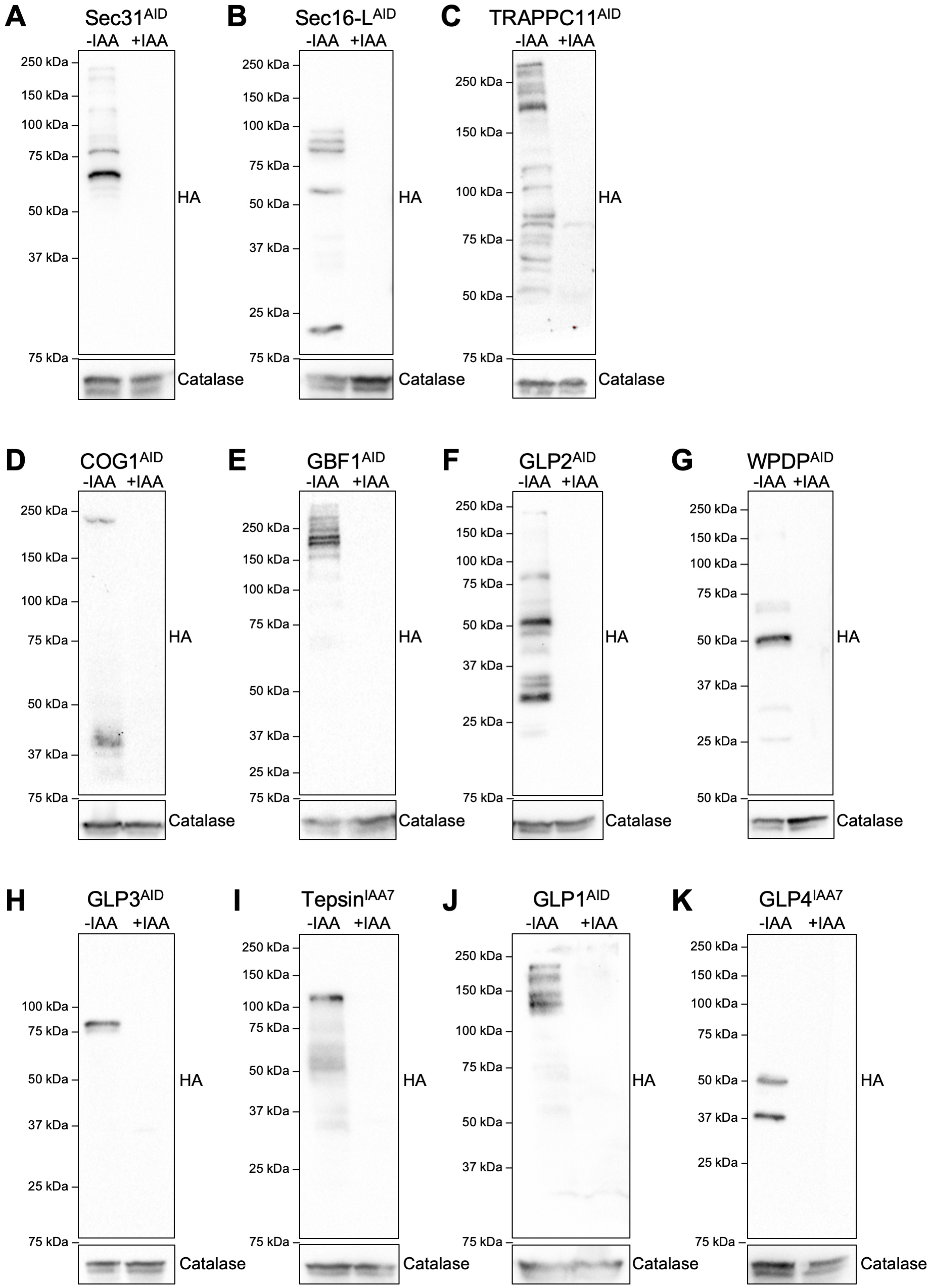

Supplement: Fig. S4 — Western blot validation for Golgi-associated proteins. [file mbio.02380-24-s0004.tiff]
