# Supplementary material for: Characterization and functional analysis of Toxoplasma Golgi-associated proteins identified by proximity labeling
Source: mBio. 2024 Sep 30;15(11):e02380-24. doi: 10.1128/mbio.02380-24 (PMC11559087; doi:10.1128/mbio.02380-24)
Supplement: Supplemental Legends — Legends for supplemental figures and tables. [file mbio.02380-24-s0005.docx]

**Supplemental Legends**

**Figure S1. Western blot validation for ULP1^AID^ and Δ*ulp1* strains.**

A) Western blot showing that ULP1^AID^ is efficiently depleted after 24 hours of IAA treatment. Catalase is used as a loading control. B) Western blot confirming loss of ULP1 in Δ*ulp1* parasites. Catalase is used as a loading control.

**Figure S2. The apicoplast and mitochondrion are unaffected by ULP1 depletion.**

A) Apicoplast morphology is unaffected by depletion of ULP1. Magenta = anti-ATrx1, Green = anti-IMC6. B) Mitochondrion morphology is unaffected by depletion of ULP1. Magenta = anti-F1β ATPase, Green = anti-IMC6. Scale bars = 2 µm.

**Figure S3. PCR verification for all Golgi-associated proteins.**

A) Diagram indicating the strategy used for PCR verification of endogenously tagged proteins. Arrows indicate the binding location of primers used. B) PCR verification for all Golgi-associated proteins.

**Figure S4. Western blot validation for Golgi-associated proteins.**

A-K) Western blot showing that all degron-tagged proteins are efficiently depleted after 24 hours of treatment with IAA. Catalase is used as a loading control.

**Table S1. ULP1 TurboID results.**

List of genes identified by mass spectrometry in the ULP1 TurboID experiment. Spectral counts are shown for each gene. “Enrichment Diff” refers to the difference between the average spectral count in ULP1^TurboID^ and control parasites. “Enrichment Fold” refers to the average spectral count for ULP1^TurboID^ samples divided by the average spectral count for control samples. GWCS = phenotype score assigned in a genome wide CRISPR/Cas9 screen (42). SP = signal peptide. #TMDs = number of transmembrane domains.

**Table S2. ULP1 immunoprecipitation results.**

List of genes identified by mass spectrometry in the ULP1 immunoprecipitation experiment. Spectral counts are shown for each gene. “Enrichment Diff” refers to the difference between the spectral count in ULP1^3xHA^ IP versus control experiments. “Enrichment Fold” refers to the spectral count for the ULP1^3xHA^ IP sample divided by the spectral count for the control sample. GWCS = phenotype score assigned in a genome wide CRISPR/Cas9 screen (42). SP = signal peptide. #TMDs = number of transmembrane domains.

**Table S3. Oligonucleotides used in this study.**

**Table S4. Raw data for quantification of plaque assays and western blot.**
